# Young People Navigating the Looping Effects of Long Covid: Exhausting Agency and the ‘Ongoing After’ of Pandemic

**DOI:** 10.1111/1467-9566.70210

**Published:** 2026-06-30

**Authors:** Tim Rhodes, Hannah Cowan, Zaira Clarke, Praveena Fernes, Sammie Mcfarland, Helen Ward

**Affiliations:** ^1^ London School of Hygiene and Tropical Medicine London UK; ^2^ Long Covid Kids Poole UK; ^3^ School of Public Health, Faculty of Medicine Imperial College London UK

**Keywords:** Long Covid, looping effect, pandemic, social life of illness, time, young people

## Abstract

Seeing beyond the COVID‐19 pandemic as an extraordinary spectacle situated in the past, we draw on qualitative research with young people to trace experiences of Long Covid as ‘looping effects’ of entangling physical and social impacts which intersect with, and extend, those of pandemic. Navigating the capacity to do ordinary things becomes highly contingent, and exhausting work, in the face of Long Covid. The physical impacts of illness, including fatigue, are materialised in altering biographical and social relationships, and managing the ‘social life’ of Long Covid is a key concern. The lifetimes of Long Covid and pandemic also feed into one another. Configurations of the COVID‐19 pandemic as a thing of the past, and social responses felt discounting of the Long Covid experience, enact ongoing illness as ‘out of sync’, and contribute as elements of exhausted agency and extended precarity. The social impacts of the pandemic looping with Long Covid become iterations without clear end; events ongoing in the living present. Understanding how Long Covid extends the ‘ongoing after’ of pandemic highlights the need for lasting social support for affected young people.

## 1 Introduction

Long Covid is an unpredictable trajectory of multiple symptoms which can involve debilitating fatigue, muscle and joint pain, shortness of breath, chest pain, cognitive impairment, post‐viral inflammatory reactions and organ damage, among other things (Greenhalgh et al. 2024; Al‐Aly et al. 2024). Estimates of prevalence are haphazard, but around 22% of adults in the UK report persistent symptoms three months after SARS‐CoV‐2 infection (Atchison, Davies, et al. 2023), as do 12.5% of young people between 12 and 17 years (Atchison, Whitaker, et al. 2023). Much attention has focused on young people's vulnerability to the mental health effects of the COVID‐19 pandemic more generally, including in relation to lockdowns, social isolation and disruptions in social, home and work life (McKinlay et al. 2022; Pearcey et al. 2023; Viner et al. 2022). Long Covid, including through its physical manifestations, intersects with and amplifies, the mental health and social impacts of the pandemic. These entanglements—of Long Covid and pandemic—create altered and precarious futures for young people (MacLean et al. 2023; Hafstad and Augusti 2021). The pandemic has been presented as a ‘threat’ to a ‘whole generation’ of young people, sometimes termed the ‘COVID Generation’ said to be ‘lost’ or ‘scarred’ by pandemic precarity (Leavey et al. 2020; UNICEF 2020). Qualitative studies focussing on Long Covid specifically suggest that the biographical disruptions of illness can be dramatic, limiting life opportunities as others move on (Maclean et al. 2023; Fang et al. 2024; K. Hunt et al. 2024).

At the same time, a key theme of qualitative work is that people living with Long Covid can feel that their lived experiences of illness are ‘invalidated’ by health service and other responses (Ireson et al. 2022; Russell et al. 2022; Maclean et al. 2023; Clarke et al. 2026). Maclean et al. (2023) suggest that Long Covid lacks ‘candidacy’, with claims of illness and patienthood ‘rejected’ or ‘diverted’, and with people ‘disbelieved’, ‘discounted’ and ‘abandoned’. Other qualitative studies note that people seeking help and support in relation to ongoing illness experience can feel ‘dismissed’ and ‘gaslighted’ (Au et al. 2022; Russell et al. 2022; Hawke et al. 2023). One key site of contestation are conflations of Long Covid with ‘mental health’ concerns linked to the crisis of pandemic that discount the physical effects of Long Covid (Ford et al. 2024; Stephenson et al. 2022; Conner et al. 2024). Conversely, there is the contested discounting of social and psychological impacts linked to Long Covid (Ireson et al. 2022; J. Hunt et al. 2022). Quantitative studies have attempted to delineate the causal temporalities of connections between the physical and social impacts of Long Covid and of the pandemic in response to symptom and diagnostic uncertainties (Nugawela et al. 2024; Stephenson et al. 2022; Engelmann et al. 2024). In this paper, we look at how young people navigate the social life of Long Covid in the ‘ongoing after’ of the COVID‐19 pandemic. We explore how the entangled lifetimes of Long Covid and pandemic feed into one another as well as extend illness beyond the multiplicity of physical symptoms alone.

### 1.1 Illness Looping Effects

The multiplicity of symptoms which make up the Long Covid and pandemic experience intermingle and cannot be pulled apart from their unfolding social relations. The lived entanglements of the physical and social effects of illness can be understood as *intra‐actions* (Barad 2007). Here, things like symptoms and embodied effects, as well as illness classifications, are not treated as stable entities but as mutually constituting of the other through their entanglements. Thus, illness is materialised, as well as made multiple, in the relational ‘becoming’ of emergent intra‐actions, rather than shaped in the intersections of distinguishable elements with identifiable causal pathways (Mol 2002). One way of thinking of illness entanglement is through ‘looping effects’. Philosopher Ian Hacking saw illness classifications and illness experiences constituting the other in a recursive fashion through their looping effects (Hacking 1995). In Hacking's work, configurations of illness experience are ‘moving targets’ that are ‘made up’ in relation to socially constructed categories. Hacking emphasised looping effects in relation to things of a ‘human kind’—how human subjectivities are shaped in socially constructed classifications—but ideas of looping effect can also emphasise the incorporation of non‐human material things of a so‐called ‘natural kind’—such as biological processes—that might be presumed more stable or free of socially constructed looping effect (Hacking 1995, 2006). Here then, looping effects broaden from a concern about the making up of *people* as effects of classifications to the becoming of *illness* as effects of material entanglements (O’Shea 2018; Barad 2007). Illness *comes to be* in the dynamic of evolving feedback loops produced in the coming together of multiple actors, human and otherwise—viruses, symptoms, bodies, discourses, ideas, diagnoses, technologies, policies, material situations and so on (Mol 2002). Importantly, looping effects are not closed or singular circuits but are ongoing in their circulations, altering the material assemblages of which they are a part as they revolve (Law 2004; Barad 2007).

Of relevance here are enactments of the problem of COVID as relatively speaking ‘small’ and ‘manageable’ in relation to young people (Conner et al. 2024). Ideas that young people are largely unaffected by COVID have infused national policies seeking a ‘return to school’ and a ‘return to normal’ (UK Government 2022; Institute for Government 2021; Parliamentary Office of Science and Technology 2021; Gurdasani et al. 2022). Narratives that downsize the problem of COVID among young people entangle with those that minimise the problem of Long Covid (K. K. Barker et al. 2022; Ireson et al. 2022; Conner et al. 2024). The lack of social currency in popular discourse regarding the severity of Long Covid, in combination with discourses that minimise the problem of COVID among young people more generally, can enact a ‘double invisibility’ (Wild et al. 2024). Patient experiences of ‘illness invalidation’ are not uncommon in the face of new and ambivalent chronic conditions which challenge medical authority (Dumit 2006; Nettleton et al. 2004).

### 1.2 Illness Lifetimes

Looping effects also draw attention to how experiences of the living present entangle pasts and futures. Chronic illness conditions loop together multiple temporalities of illness experience (Green and Lynch 2022). Events configured as past fold into the present; as ‘living’ in the here‐and‐now; extended in their looping effects of past–present–future (Deleuze 1994; Barad 2010). In intra‐actions, time and space and objects loop inseparably together, with scenes of experience circulating as ‘unending iterative reconfigurations’ (Barad 2010). Illness events, including collective ones such as pandemics, do not have clear cuts of ‘before’ and ‘after’ in a linear progression, but ‘live on’, in their enactments in social life producing multiplicities of lifetime (Barad 2010; Berlant 2011).

The problems of COVID and the COVID‐19 pandemic are increasingly configured as things of the ‘past’ or as ‘over’. In the UK, for example, the recent COVID‐19 Inquiry performed a narrative of *looking back* at the pandemic as a *past event* to learn *lessons* for the *future*. As noted in UK Government responses: ‘The impact of the COVID‐19 pandemic was unprecedented in modern memory […]. It is our duty to learn the lessons of this Inquiry and be better prepared for the next pandemic’ (UK Government 2025). Whilst noting COVID as a continuing threat, WHO declared an ‘end’ to COVID‐19 as a global emergency in 2023 (United Nations 2023). In discursive enactments of the COVID‐19 pandemic as past and as less urgent, the ongoing social and material effects of the pandemic, and of COVID, including as they affect ordinary life and futures, can get downplayed. The condition of Long Covid, and embodiments of ongoing illness, can present as matter that is ‘out of sync’ with an imagined COVID past.

Enacting the pandemic as an event of the past is an *imagined synchronisation* which simplifies the multiplicity of its lifetimes (Wigen 2022). Framings of ‘crisis’—such as the COVID‐19 pandemic—tend to simplify their diffuse multidimensional temporal and spatial elements into singular containable configurations (Roitman 2014; Wigen et al. 2021; Rhodes 2025). This is a process which appears to help when navigating uncertainty and galvanising collective action in the face of emergency (K. Barker 2012; Wigen et al. 2021). Cultural theorist Lauren Berlant encourages an alternative view of crisis which not only enacts such events as abrupt, extraordinary and spectacular, but which attends to the ‘ongoingness’ and ‘ordinariness’ of crisis in everyday life; processes which do not present as sudden aberrations but which are chronic and diffuse in their habituations, and which produce a ‘latent’ and ‘exhausted’ agency over time (Berlant 2011). This alternative view of ‘ordinary crisis’ extended in social life helps to see the ‘ongoing after’ of events otherwise enacted as past.

## 2 Methods

We interviewed 72 young people aged between 15 and 25 years in the UK. Interviews were undertaken between January–November 2024, with follow‐up interviews (*n* = 30) undertaken between May 2024–February 2025. The study included: young people who received a diagnosis of Long Covid from a healthcare professional (*n* = 23, of whom 19 were followed‐up); young people experiencing persistent COVID‐19 symptoms and self‐diagnosed as Long Covid (*n* = 15, of whom 6 were followed‐up) and young people who described persistent and ongoing health effects related to SARS‐CoV‐2 without attributing them to Long Covid (*n* = 16, of whom 5 were followed‐up). The study also captured young people's wider health and wellbeing experiences linked to the pandemic (*n* = 18, none of whom were followed‐up). For our purposes here, we draw on interviews with the 54 young people medically or self‐diagnosed with Long Covid (*n* = 38) or living with ongoing symptoms linked to SARS‐CoV‐2 (*n* = 16), of whom 30 were followed‐up at around six months. Participants were recruited in collaboration with the REACT study (*n* = 44) led by Imperial College (Atchison, Whitaker, et al. 2023), the charities Long Covid Kids (*n* = 9) and Long Covid Support (*n* = 3) and community organisations working with young people (*n* = 16). Ethical approval was granted through the London School of Hygiene and Tropical Medicine Observational Research Ethics Committee and via the Research Ethics Committee of Imperial College. We engaged young people in the data generation stages of the project via a series of eight consultation sessions, and in the data analysis stages through 15 workshops with a peer collaboration group of 10 young people with experience of Long Covid.

Of the 54 young people experiencing Long Covid and ongoing symptoms, 21 were aged between 15 and 18 years at the time of their first interview, 9 were aged 19–21 years and 24 were aged 22–25 years. Most (*n* = 34) described themselves as female, 17 as male, one as non‐binary trans masculine, one as non‐binary and one chose not to describe their gender. We note that our study includes a higher proportion of White female participants (24). Some described themselves experiencing neurodiversity (*n* = 20) or additional disabilities (*n* = 6). In interviews, around 15 described experiencing symptoms of Long Covid since 2020 (for around four years), 15 since 2021, six since 2022 and four since 2023. Fourteen described ongoing symptoms between 2020 and 2023 but saw these as past. Most (*n* = 36) were therefore navigating their Long Covid through the time of COVID‐19 lockdowns in the UK (2020–2022), and since, with the minority (4) first experiencing Long Covid after 2023, a period characterised by policies seeking a ‘return to normal’ in the ‘after’ of pandemic (UK Government 2022; Institute for Government 2021).

Interviews were loosely structured conversations, lasted around 1 hour, with all but one online. Core questions were circulated in advance and there were options for breaks in conversation and re‐scheduling. Two participants were joined by a family member. We sought to generate accounts of experience in relation to the physical and social effects of Long Covid and COVID‐19 over time. We developed a descriptive coding framework through a series of six workshops open‐coding 18 transcripts (Charmaz 2006), which then formed the basis of coding in NVIVO across all transcripts. NVIVO coding was undertaken iteratively as data were produced, and was workshopped in ongoing team discussion and with a peer collaboration group of young people with experience of Long Covid. Pseudonyms are used throughout.

Our analytical aim here is to trace emerging threads in narratives of social life affected by Long Covid entangling with pandemic. By social life, we refer at once to how the object of illness of Long Covid is produced in social relations and how these enactments recursively affect those social relations which are incorporated in experience going forward. We wish to draw attention to how ongoing illness experiences are extended in their social effects, beyond physical embodied symptoms alone, including as alterations which produce an unsettled living present and sense of precarious future. Our analysis draws on the ideas of crisis ordinariness (Berlant 2011), intra‐action and entanglement (Barad 2007) and looping effect (Hacking 1995).

We note that young people's accounts entangle together enactments of illness as ‘past’ (when looking back to describe events) with illness as ‘present’ and ‘ongoing’ (when describing events in the here and now). This inevitably makes distinguishing past from present in illness experience complex, accentuating how temporalities of illness are situated relationally in the living present (Green and Lynch 2022). Our analysis here does not attempt to distinguish accounts of illness as a function of calendar time but explores how temporality is enacted in relation to how the lifetimes of Long Covid and the COVID‐19 pandemic entangle. We draw attention to accounts of the physical and social looping effects of ongoing illness as non‐linear and unpredictable. We indicate where accounts draw on baseline (Interview 1) and follow‐up (Interview 2) data.

## 3 Analysis

We draw out three intersecting themes from the interview narratives: extraordinary struggles of the ordinary; altered social life and exhausting agency. Taken together, the experience of Long Covid is enacted as a looping effect of physical, social and temporal entanglements. We bring our findings together through the analytic of the ‘ongoing after’ to trace how Long Covid intersects with the COVID‐19 pandemic to extend illness in social life, including without clear end.

### 3.1 Extraordinary Struggles of the Ordinary

Emma, a 17 year old who has been grappling with diagnosed Long Covid since she was 13, accentuates Long Covid as ‘life changing’, drawing attention to things that can no longer be done:

Who I was prior to Long Covid and who I am now are quite different things […].Before the pandemic, it was great, I was happy, I was healthy, I was living a normal life, I was just a normal kid […]. And then when I got ill, that’s when like everything changed […]. I went from being able to go to school, being able to play tennis and stuff, to being virtually bed bound […]. I don’t leave the house. I can’t leave the house other than to go to hospital appointments in a wheelchair, and even then it’s hard […]. [It] is tumultuous, up and down, hellish […]. It has been a real ordeal, life changing, life upending, like nothing has remained the same […]. I’m now 17, and it’s been a rollercoaster since I was 13.

(Emma, Aged 17, Interview 1)

Young people describe the physical manifestations of Long Covid as multiple and fluid. Rose's medically diagnosed long OVID for instance, has included ‘cold, flu symptoms’, ‘migraines’, ‘bad stomach issues’, ‘nerve pain’, ‘muscle spasms’, ‘really bad nosebleeds’ and ‘being exhausted’. Ayesha's Long Covid, self‐diagnosed, has included ‘fevers’, ‘vomiting’, ‘diarrhoea’, ‘random spikes in my temperature’, sleep problems, breathing difficulties, ‘brain fog’ and being ‘really tired’. These multiple physical symptoms entangle in unpredictable ways. As Emma says: ‘I think every single day is different, you know, symptoms come and go’. It's like ‘Whac‐A‐Mole’.^1^ This has made living day‐to‐day highly contingent: ‘You can never really make plans with anything far in advance, because you just never know how you'll be feeling’ (Emma, Aged 17, Interview 2).

The everyday impacts of Long Covid, especially through the exhaustions of physical and mental fatigue, make doing ordinary things extraordinary. Rilee, who was 25 at the time of interview, described her illness as ‘lingering’ as well as a ‘domino effect’ of multiple symptoms that built into an intensity that became ‘insane’ and that made doing ordinary things, such as walking, extraordinary:

It was like a domino effect, different things keep occurring in my body […]. I couldn’t get out of bed to go to the doctors […]. I knew I had had Covid, so I knew that the symptoms that I was experiencing was lingering on from that, but I wasn’t aware of like how bad Long Covid could be […]. I felt like I was *dying* […]. It was the most insane experience I’ve ever gone through […]. When I was bedbound, I genuinely thought that my body was like slowly dying […]. The biggest symptom is like a really severe fatigue, so it’s like trying to walk under water when you have like loads of waves trying to push you different directions and you’re trying to walk straight. Or like you have a hangover, you have the flu, you have all these different things all at the same time, like it was just so *intense*.

(Rilee, Aged 25, Interview 1)

Rilee moves between past and present tense in her accounting of illness; as something that ‘was’ (when looking back at an ‘intense’ and ‘insane’ illness experience) as well as still ‘is’ (ongoing severe fatigue and symptoms that ‘keep occurring’). Because of fatigue and heart difficulties, Rilee says that ‘being able to actually sit upright, and things like that, like really simple basic things that I never used to think about, are like huge achievements to me now’. Similar to Emma and Rilee, Ryan's Long Covid is also physically draining: ‘I'm exhausted, and if somebody asks me to do something when I don't have the energy practically to breathe sometimes, I'm going to snap’. Those whose experiences of illness following SARS‐CoV‐2 initially presented in less physically dramatic ways, also see their COVID‐19 as an ongoing day‐to‐day struggle. For instance, Amy's SARS‐CoV‐2 infection was followed by extreme fatigue, which has persisted, as other symptoms, such as chest tightness, have fallen away. Amy is uncertain about how to make sense of the lasting effects of SARS‐CoV‐2, and she has not sought a medical diagnosis of Long Covid, but how these impact on her capacity to do ordinary things are no less insignificant. Her account, similar to Rilee's, entangles the living present with past fatigue:

I had it for months, this extreme fatigue, where I just couldn't do anything. I couldn't do anything without like then needing to sit down and have a nap […].I do feel like I'm not the same person that I was before I had it […]. Since having it, like I've just, it'll kind of get to like 10am and I'll crash, and then like I'm really struggling like with my day.

(Amy, Aged 25, Interview 1)

There are two points to highlight here. First, the extended physical impacts of Long Covid, and of SARS‐CoV‐2 for some, make the ordinary extraordinary; an incorporation of crisis into the everyday (Berlant 2011). One feature of young people's accounts here, is how embodied ongoing illness feels ‘out of sync’ with the ordinariness of being young: ‘Obviously it upsets me with like my heart and everything, because certain things like I struggle with, like going up flights of stairs, and it's just rubbish […]. I'm young, I shouldn't be having to experience things like that’ [Isla, Aged 18, Interview 1]. Natalia's Long Covid, for instance, makes her feel out of place, at once physically and socially: ‘I'd only just turned 18, so all my friends were out and everything, and then I was in hospital. […] I was the only young person in there. […] I just wanted to find someone who was young, like my age, who had it, to see how it affected them’ (Natalia, Aged 20, Interview 1). Natalia's feeling of being out of sync was met with her yearning to know that she was not the only extraordinary one, that perhaps there were others who ‘were scared to go out’ and who ‘felt like socially they were like a bit awkward’.

Second, the ordinary things that become ‘huge achievements’ are highly contingent on negotiating reduced energy potentials, and this is *work* that is not only unpredictable and experimental but itself exhausting. For instance, Fern, who is 20 years old, emphasises in her follow‐up interview how the looping exhaustions of the mind and body are in delicate balance. Pacing day‐to‐day, for Fern, is ‘brain or body, or neither’. She has learnt, through trial and experience, to set ‘quite strict like time limits now’ because ‘things need to be a bit more spread out’. She realises that she needs to be ‘really careful, even more so now, in terms of energy’, as the care appointments she is now seeking are physically demanding of energy, which means that she is ‘not as able to do as much brain stuff’. Jack too, like others in our study, draws attention to managing reduced energy potentials as a difficult balancing of mind with body, of physical fatigue with mental exhaustion, which orientates around ‘trying to save as much energy’ as possible while doing things, and which itself is exhausting work that ‘takes energy’. This is a question of doing less and balancing what can be done: ‘If I do have to socialise […], it's just harder, and it takes more energy, and then I find I can't do other things’ (Jack, Aged 17, Interview 1).

### 3.2 Altered Social Life

The physical impacts of Long Covid are inseparable from how they are lived in the everyday; what we describe here as the social life of Long Covid. As with Natalia and Jack above, young people's accounts draw attention to the social life of Long Covid exhaustions and how these alter the ordinariness of social interactions, relationships and futures.^2^ The social life of Long Covid not only extends illness beyond its multiplicity of physical symptoms but also loops together with past and ongoing experiences of the COVID‐19 pandemic.

Noah was 16 at the time of his interview. His Long Covid entangled with pandemic lockdowns to extend the isolating impacts of altered everyday relationships, including into the future, as meeting new people also became difficult. He describes a loss of social contact as an isolation which has been made ongoing yet timeless:

It was a lot of monotony […], doing the same thing every day for weeks on end, not really able to go out […]. I spent a lot of the time in my back garden, you know, spending time with my friends online, just trying to sort of keep myself, yeah, sane […]. It was so bleak because it was not, I wasn't really getting better, it was just sort of the same, you know, timeless. My childhood was pretty much just slipping away, and I couldn't do anything about it […]. It severely impacted my sort of time to make friends at [secondary school] with new people […]. For a long time I was very isolated […]. No one had really heard of me […]. We had Covid, and then I had Long Covid, you know, just sort of a blip where no one really knew I existed.

(Noah, Aged 16, Interview 1)

Noah's account accentuates the monotony of ‘not doing’, and of doing the same things for weeks, as a form of lost and latent agency; a situation in which his sense of normal life and trajectory was slipping away but where there was little capacity to do anything about it. Noah situates his Long Covid as something that will hopefully end, saying that the ‘tunnel might be long, but there is light at the end of the tunnel’. But in the meantime, his Long Covid presents as an ongoing diminishing of agency in the social world that extends the isolating effects of the pandemic; an absence of opportunity to exist in relation to others, as others are moving on.

Noah is not alone in this description of social detachment extending in time. Sophia is 17, and has been living with medically diagnosed Long Covid for four years. As her Long Covid has extended in time, her friendships slowly disappeared. She says that ‘I've basically lost everyone I knew in person before’. She feels forgotten: ‘People have pretty much forgotten about me, that I exist’. Emma similarly talks of the slow disappearance of friends as a collateral of Long Covid ‘I used to have like a lot of friends in my school, and obviously when I got so ill, they kind of disappeared’ (Emma, Aged 17, Interview 1). Others were made to feel absent, ‘kicked out’, as if matter out of place: ‘Over the course of half a year I went from being in that friend group, then I got Long Covid […]. Within half a year from that point, the year after I got it, then they kind of kicked me out of the friend group’ (Jack, Aged 17, Interview 1).

Although entangled, it is important to recognise that the social effects of the pandemic and Long Covid are not the same.^3^ Long Covid is presented as ‘more than’ the effects of pandemic in the ‘crisis ordinariness’ of altering of social life (Berlant 2011). Freya, whose Long Covid has involved hospital stays, acknowledges the isolating impacts of lockdowns among her peer group, but sees her situation as qualitatively different: ‘“You're not going through *this* [Long Covid]. You all still seem to know each other. You still seem to be *doing things*”. I would have loved to be in their position’ (Freya, Aged 23, Interview 1). The ongoing yet unpredictable effects of chronic fatigue linked to Long Covid, combined with brain fog among other symptoms, enact a particular strain on social relationships, with lasting effects. As Hazel says, people find it difficult when plans are made and then cancelled at the last minute, and this becomes difficult to manage over time: ‘I like finally felt able to make plans with people, and then like it came around to it, and I didn't want to let people down, but I didn't feel up to it. That definitely has been like quite straining on my relationships.’ (Hazel, Aged 20, Interview 1).

Isabella accepts that ‘I have to really pace my life now’, and that this involves ‘putting boundaries up’ to minimise, or retreat from, social contact (Isabella, Aged 18, Interview 1). At her follow‐up interview, Taylor says she has had to find a balance in ‘sacrificing my life’ in her attempts to sustain disappearing friendships following her Long Covid and ‘sacrificing people’ who she came to realise were hopes of friendship that she had to let go. Emma draws attention to social interactions made uneasy since Long Covid as they lost their taken‐for‐granted ‘know how’ of the untroubled before. She remarks that ‘My old friends definitely didn't know how to sort of act, and that definitely like really changed our relationship, and pretty much ended it I'd say’ (Emma, Aged 17, Interview 1). For Emma, the combined difficulties of managing exhaustions (‘Even going out I'm wondering if I'm going to crash when I'm out’) and the risks of re‐infection (‘My anxiety is targeted around Covid, and around my health, and around getting ill again’) produce a living present of precarity. At her follow‐up interview, she says she has found herself retreating from social life, and says ‘I have virtually lost everything that I previously like held as my identity’. The ‘ongoingness’ of her illness is a looping together of physical and mental health effects which fold into the other. She says Long Covid ‘has caused anxiety for me, and depression, and I don't think you could separate them’. She says her struggle with ‘depression’ is because ‘I'm constantly in the house, like feeling really rubbish every day, which takes its toll after 3 years’.

Kelly, who is 23 years old, has been grappling with fatigue and brain fog for two difficult years. She reflects on how her brain fog entangles other things, including the trauma of an emergency hospital stay, to produce anxiety, as well as a sense of reduced agency in ordinary social interactions. On her return to university after having had 2 years away, Kelly says she has ‘not made a single friend’. She asks of herself ‘Why can't I do this?’. She says: ‘I just can't speak to anyone, I can't put myself out there, I struggle to leave the house, like it's just unbelievable’. Narrating Long Covid as a biographical turning point, Kelly sees a ‘stark difference between me before and after’. Her account emphasises that any one symptom or effect—such as brain fog—is never separate but comes to affect ‘every aspect of life’ through the momentum of their entanglements. Whereas she was ‘always very like confident and talkative at school’, she says that ‘now I'm just not at all’. She has become ‘so anxious’ in social interactions, because of her stutter, and because of her ‘absolutely terrible’ memory where her ‘brain won't absorb any new information’. Kelly says she is ‘really struggling’ because her Long Covid has ‘affected me in every aspect of my life […], like work, study, friends, family, like literally everything’. Kelly's sense of self and social life retreating since the pandemic has been amplified by Long Covid. She says she no longer wants to ‘put myself out there’ instead opting ‘to just sit in the background and blend in’. She has become ‘this completely different person’. This altering of self and life is not only a loss of what was before but of anticipated future. When asked about the future, she says the following:

I try not to think about the future, because it just scares me. I think if you'd asked me that before Covid, I would have reeled off like a list of things that I wanted to do and I wanted to achieve, and what I wanted to be. And now, I just don't know.

(Kelly, Aged 23, Interview 1)

Long Covid can present then, as an altering of social life as it was before, or as an ‘ending’ of how it was anticipated; a sense of desynchronisation that is made manifest in how COVID and the pandemic ‘live on’ for those navigating ongoing illlness. The pandemic is not simply a figure of the past but embodied in‐the‐now of ongoing illness, as in the case of those experiencing a pervasive sense of reinfection risk:

I was paranoid like for about a year. I would, anywhere I would go, mask, because at that time restrictions were calming down, you didn’t actually have to wear a mask and everything. But to have hand sanitizer with me everywhere, and mask everywhere, wipes everywhere. As soon as I got home, I would shower. I couldn’t have any germs. If anyone coughed next to me, oh my God, I was literally panicking so much. […] I was just panicking all the time.

(Natalia, Aged 20, Interview 1)

Natalia's description of this ongoing state of unease as ‘paranoia’ is telling; for it sets apart her experience as something different, at odds, to the anticipated ‘normal’. It is the ongoingness of Natalia's illness that makes her feel ‘out of place’ socially and ‘out of sync’ temporally in relation to imaginaries of the pandemic as passed. Natalia becomes distanced, and more on her own, as time extends. Wearing face masks materialises this disjuncture: ‘You're the only one wearing a mask, and even like, if I was travelling with friends, they wouldn't wear masks, and I'd just be so paranoid’ (Natalia, Aged 20).

This sense of detachment—in relation to imaginaries of the pandemic past and of the social world of before—is, therefore, at once an entanglement of the living present; a looping in of the ‘pandemic now’ as an element of ongoing illness. Emma, for instance, accentuates how circulating notions of pandemic as past can loop into the living present to energise its unending presence. At her follow‐up interview, Emma says that as the time of pandemic for others has past, she is made to feel ‘stuck’, immobilised by ongoing illness. She says ‘It kind of feels like the world has moved on, and we're kind of still stuck in the pandemic where everyone was isolating’. She presents her life with Long Covid as ‘out of sync’. Her world living with Long Covid has become a ‘lonely place’: ‘Every day that goes by, people kind of get further distanced’. Three years on, she is ‘still virtually isolating’. She comments that for others the world ‘just seems normal and everyday’, as it was before. But for her, there is no ‘going back’, ‘even once I recover’, as her future has already altered: “It's definitely changed how I look at things’.

### 3.3 Exhausting Agency

Navigating altered social life and relationships is not easy work. This is social interactional work that itself can become exhausting in the face of reduced energy potentials and diminished agency affected by being made ‘out of place’ and ‘out of sync’ by the spacetime of ongoing illness (Barad 2007; Berlant 2011).

Taylor is 18, and trying to find ways to manage her medically diagnosed Long Covid. A tactic of hers is to ‘hide’ her Long Covid, because she has felt herself to have become an ‘inconvenience’ to others:

I feel like people get annoyed at me, because I’m constantly saying like, ‘Oh my God, I feel so exhausted today, or I can’t concentrate very well, or I’ve gone dizzy, or I feel nauseous, or my stomach hurts. And when I say this to people I feel like they’re like’, Why do I need to know this information, I don’t really care.

(Taylor, Aged 18, Interview 2)

Taylor, whose autism is also described as having a bearing on how she manages social interactions, has learnt to mask to others how she feels. Rather than giving ‘depressing answers’ because ‘our lives at this time are depressing us’, she says ‘I've got to make myself seem kind of happy, and like I am coping well, so I just now tend to say “I'm fine, I'm doing great”’. Similar to Taylor, Rilee also says she ‘struggles with telling people that I have [Long Covid]’. Rilee cannot help but see her Long Covid as ‘my own weakness’. She says that ‘I don't want to be seen as vulnerable, or targeted because I'm vulnerable’. She is conscious that some friends do not appreciate ‘the longevity of it’, and ‘don't see the fatigue as like a serious symptom’. This makes Long Covid difficult to manage over time, and similar to Taylor, Rilee says that ‘I have gotten rid of more friendships than I've gained, just because I've had to notice which ones were not supportive anymore’.

Jack presents his Long Covid as a problem of exhaustions, accentuating how the efforts to manage the reduced energy potentials of mind and body perversely contribute to the lessening of his energy reserves. Similar to Taylor and Rilee, Jack finds the social interactional work of navigating his Long Covid challenging and draining. He gets ‘tired when trying to explain’ his Long Covid. It would help, he says, if people understood Long Covid as ‘actually a thing’, because ‘it is easy to dismiss’, and it would help if people ‘know about Long Covid and what I had to do’. The pervasive need to account for Long Covid exhaustion in the face of push‐back and misunderstanding ‘takes a lot of energy’. By the time of his second interview, Jack, similar to others, has found himself retreating from social life as a means to managing exhausted agency. In consequence, he says his ‘social existence now is definitely different to what it was before’. Jack says that over time he has learnt to become ‘happy to be by myself’ and that his altered social life ‘bothers me a lot less than it used to’. As time has moved on, he says, ‘I've just got used to it now’.

Enactments of the pandemic as a shared event of the past can minimise ongoing illness experience, making it more exhausting to manage. Navigating the ongoingness of Long Covid into everyday social interactions that are infused by imaginaries of the pandemic as past create temporal collisions in the ‘absent–presence’ of illness: ‘Everyone wants to pretend it didn't happen, and everyone wants to move on’ [Theo, Aged 25, Interview 1]. Young people navigating Long Covid say that their peers living through the time of pandemic may not grasp, or want to know, what the particularities of Long Covid are like. Long Covid is not only ‘out of sync’ with the pandemic past but with *young people*:

Even now, when I tell people I’ve got Long Covid, they’re like, ‘But you’re so young!’

(Isabella, Aged 18, Interview 1)

I definitely don’t think people my age really understand Long Covid, because I think they just think sometimes that you make it up, or because sometimes it’s an invisible illness […] You mention Long‐Covid, they kind of go, ‘Ooh, you look perfectly healthy, what are you on about? There’s nothing wrong with you!’

(Taylor, Aged 18, Interview 2)

For Natalia, whose experience of COVID hospitalisation was ‘traumatising’ and without clear end (‘I'm still not over it’), there is a stark contrast drawn between the weight of ongoing illness and the lightness afforded by narratives which belittle the seriousness of COVID and the pandemic among younger people:

For my generation, I feel like people just see it as a joke, do you know what I mean? I don’t think there is any support at all, really, for young people who had it. […] A lot of people thought they wouldn’t be affected as bad because, obviously, we were told because we were young that we wouldn’t be affected from it. […] So, I just think they just kind of saw it as a joke, to be honest.

(Natalia, Aged 20, Interview 1)

In an atmosphere of disappearing pandemic, the social interactional work of navigating Long Covid demands ‘extra effort’ to be ‘seen’. Taylor says that ‘You have to put that extra effort to see if they can see how it's affecting you’ (Taylor, Aged 18, Interview 2). She further explains that in the absence of ‘presenting with physical evidence’—through symptoms or mask wearing—the Long Covid *experience* is made invisible: ‘If they can't see it’, they ‘just don't care’, ‘it doesn't exist’. Sophia accentuates how the extraordinary work to navigate ongoing illness into existence loops across multiple social fields. In her first interview, she describes her body as ‘in fight or flight mode all of the time’. A big part of this ‘flight or fight’ is navigating the social life of illness, in public space, among friends, at home as well as at school. She says: ‘I go out in public, and people are like “Oh, why are you wearing a mask, you know, masks don't work, she is just mentally ill, that's why she is wearing it”’. At school, she says that others act as if COVID is in the past and no longer a problem: ‘It's like I'm running from a bear all the time, but there is no bear, and it's my classmates’. At her second interview, she says that home has also become a day‐to‐day challenge: ‘I had to try and explain to my dad, while literally not even being able to sit up, like what he needs to do […]. He doesn't truly understand’. Sophia highlights the invalidation of her illness experience in the face of others' questioning reactions: ‘Covid is still a thing. […] They don't want to accept the fact. […] We are not faking it’ (Sophia, Aged 17, Interview 1).

The interactional work to navigate Long Covid is an agency ‘in the balance’ given how efforts to act threaten their own limited energy potentials. When efforts to survive the constraints of ordinary life perversely contribute to those constraints, a situation of exhausted agency is enacted (Berlant 2011). Long Covid enacted as insignificant or doubted, and configurations of COVID and the pandemic as past, loop in as elements of the exhausted agency of ongoing illness.

## 4 Conclusions

Our analysis of Long Covid as a looping of social and physical effects sees how illness is extended in social life. Configurations of COVID and of the COVID‐19 pandemic as past, and social responses discounting of Long Covid experience, enact ongoing illness among young people as ‘out of place’ and ‘out of sync’, and contribute as elements of exhausted agency and extended precarity. Via the ideas of Berlant (2011), we draw attention to the ‘crisis ordinariness’ of Long Covid in the ‘ongoing after’ of pandemic for affected young people.

### 4.1 Looping and Multiplicity

Despite growing appreciation of Long Covid as multifactorial (Greenhalgh et al. 2024; Al‐Aly et al. 2024), there is limited appreciation of how the multiple physical effects of illness entangle in social relations (Mol and Hardon 2020). A failure to see the variable looping effects of Long Covid, or to appreciate the social life of illness, reproduces an approach which unhelpfully separates out, in time and space, the biomedical conditions of illness from its experience. Our analysis emphasises the multiplicity of Long Covid as an emergent intra‐action of entanglements in social relations (Barad 2007). We have drawn particular attention to the ‘ongoingness’ of illness as a contingency of constraints on agency affecting the capacity to do ordinary things. Long Covid fatigue, among other symptoms, and the efforts to navigate these, combine as well as feedback in their looping effects to produce an ‘exhausted agency’ (Berlant 2011). We have also drawn attention to how seemingly separate things in the ‘ordinariness’ of illness experience unavoidably trace, through their looping effects in social relations, to larger and more complex arrangements. Examples include how physical illness entangles inseparably for young people in social life and with mental health concerns, and how past experiences, including of pandemic, extend illness in the living present. This is why we see Long Covid as intra‐acting with pandemic, where the pandemic ‘past’ loops with illness of ‘now’, and where the ‘extraordinariness’ of big events such as pandemics *live on* and *out last* their crisis framings in the ‘crisis ordinary’ (Berlant 2011).

### 4.2 The Ongoing After

Our analysis points to how illness is ‘made long’ in social life. Rather than enacting the COVID‐19 pandemic as an aberration and spectacle situated in the past, we have seen how Long Covid entangles the pandemic, with other past experience, as elements in everyday assemblages that extend illness in social life going forwards. Our analysis shows that enactments of the pandemic as a thing of the past come into collision with embodiments of ongoing COVID, and are felt as invalidating, enacting ongoing illness experience as matter ‘out of place’ and ‘out of sync’. The discounting of Long Covid contributes to the extraordinary struggle of the ordinary that young people face, looping as an element of exhausted agency (Berlant 2011). The efforts that young people make to enact COVID‐19 and the pandemic otherwise—as *not past, nor over*—are pervasive and wearing, and create feedback loops of their own in the ‘ongoingness’ of illness. Many young people find themselves retreating in social life as part of their efforts to preserve agency and navigate the ‘crisis ordinariness’ of Long Covid, an altering which also affects future life potentials. Karan Barad cautions against the ‘presumption of erasure’ in configurations of things as ‘past’ to accentuate the ‘ghosts we encountered in the flesh as iterative materialisations’ which reconfigure ‘what was, and what comes to be’ (Barad 2010, 264). The ‘ongoing after’ suggests an event *ongoing*, an iteration of after *without clear end*, an unending *presence*. We find that Long Covid is not only extended in its physical manifestations but in how these loop into altered social life, in which the ‘pandemic past’ is also made present, to unsettle what has been as well as what might become.

### 4.3 Invalidations and Desynchronisations

Research emphasises that young people affected by Long Covid and by the COVID‐19 pandemic have felt that their voices have not been heard (Maclean et al. 2023; Baz et al. 2023; Wild et al. 2024). Enactments of the pandemic as past, in combination with social responses that challenge the severity or lasting impacts of Long Covid, fold into the living present to invalidate ongoing illness experience as if ‘out of sync’. Mainstream models of science have pinned their hopes on resolving casual and illness classification uncertainties by delineating how different physical and social elements of Long Covid and the experience of pandemic interact temporally, including in relation to mental health (Nugawela et al. 2024; Stephenson et al. 2022). Our analysis alternatively suggests ‘staying with the trouble’ (Harraway 2016) of unfolding looping effects to trace how the social life of illness is materialised in everyday practices. Looking beyond the biomedical mainstream to appreciate the social life of Long Covid demands that we listen, respect and act in relation to accounts of experience rather than contribute to their invalidations and desynchronisations.

### 4.4 A Social Life of Care

Understanding Long Covid as a diffusion of looping effects is a challenge for fragmented care services linked to organ‐focused disease‐based understandings (Greenhalgh et al. 2024; Mol and Hardon 2020). Previous research highlights the fragmentations and fragilities of health care for young people affected by Long Covid (Wild et al. 2024; MacLean et al. 2023; Clarke et al. 2026). In Berlant's account of the ‘crisis ordinary’—conditions which habituate precarity and disadvantage, thereby undermining efforts to transform these—social life can become, over time, a ‘condition of being worn out by the activity of reproducing life’ (Berlant 2011). In this broader account of late capitalist life, exhausted agency is about ‘getting by’ in the ‘impasse’ of ‘survival time’. There are some parallels here with the stories of loss, reduced agency and discounted future told by some young people living with Long Covid. This leads us to conclude that a social life of care is not made up in reflex crisis responses, nor focused on Long Covid and COVID‐19 alone, and it need not give priority to technological and biomedical solutions in response to particular symptoms. Rather, it is a lasting and everyday care. At a time when the COVID‐19 pandemic is increasingly enacted as ‘in the past’ or ‘over’, and when Long Covid care and pandemic initiatives are being rolled back as if no longer required, young people navigating Long Covid emphasise the need for ongoing and entangled social support.

## Author Contributions

**Tim Rhodes:** conceptualization, investigation, funding acquisition, writing – original draft, methodology, writing – review and editing, formal analysis, supervision, project administration. **Hannah Cowan:** investigation, writing – review and editing, methodology, formal analysis, data curation, conceptualization, project administration. **Zaira Clarke:** investigation, writing – review and editing, data curation. **Praveena Fernes:** investigation, writing – review and editing, data curation. **Sammie Mcfarland:** writing – review and editing, funding acquisition, investigation. **Helen Ward:** funding acquisition, investigation, writing – review and editing.

## Conflicts of Interest

The authors declare no conflicts of interest.

## Data Availability

The original data that support the findings of this study are not publicly available because of privacy or ethical restrictions.

## Endnotes

^1^
Whac‐A‐Mole is a game found in amusement arcades in which players use a mallet in an attempt to bash moles back into their holes as they pop up randomly.

^2^
We acknowledge that the social life of Long Covid extends further, for instance, in the relations of family, work, education and so on (MacLean et al. 2023; K. Hunt et al. 2024).

^3^
In feedback among young people living with Long Covid as part of our peer collaborator group analysis workshops, including in relation to the drafting of this paper, young people wanted to make clear that the scale and intensity of the impacts of Long Covid on their capacity to navigate ordinary life and future were of a different order than those precipitated by pandemic and related lockdowns.
